# RECORD-4 multicenter phase 2 trial of second-line everolimus in patients with metastatic renal cell carcinoma: Asian versus non-Asian population subanalysis

**DOI:** 10.1186/s12885-018-4091-5

**Published:** 2018-02-17

**Authors:** Lin Yang, Anna Alyasova, Dingwei Ye, Antonia Ridolfi, Luca Dezzani, Robert J. Motzer

**Affiliations:** 1 National Cancer Center/Cancer Hospital, Chinese Academy of Medical Sciences and Peking Union Medical College, 17 Panjiayuannanli, Chaoyang District, Beijing, China; 2 Prevoljskiy Region Medical Centre, Ilinskaua St, N, Novgorod, 603109 Russia; 3 Fudan University Shanghai Cancer Center, 220 Handan Rd, Yangpu, Shanghai, China; 4 Novartis Pharma S.A.S, 2 Rue Lionel Terray, 92500 Rueil-Malmaison, France; 5 Novartis Pharmaceuticals Corporation, 1 Health Plaza, East Hanover, NJ 07936 USA; 6 Memorial Sloan Kettering Cancer Center, 1275 York Ave, New York, NY 10065 USA

**Keywords:** Asians, Everolimus, Ethnicity, Clear-cell metastatic renal cell carcinoma, Sequence

## Abstract

**Background:**

RECORD-4 assessed everolimus in patients with metastatic renal cell carcinoma (mRCC) who progressed after 1 prior anti-vascular endothelial growth factor (VEGF) or cytokine and reinforced the clinical benefit of second-line everolimus. Because of the high percentage of patients from China enrolled in RECORD-4 (41%) and some reported differences in responses to certain targeted agents between Chinese and Western patients, this subanalysis evaluated outcomes in Asian versus non-Asian patients.

**Methods:**

RECORD-4 enrolled patients with clear cell mRCC into 3 cohorts based on prior first-line therapy: sunitinib, other anti-VEGF (sorafenib, bevacizumab, pazopanib, other), or cytokines. Patients received everolimus 10 mg/d until progression of disease (RECIST, v1.0) or intolerance. Primary end point was progression-free survival per investigator review. Data cutoff was Sept 1, 2014.

**Results:**

Among Asian (*n* = 55) versus non-Asian (*n* = 79) patients, 98% versus 84% had good/intermediate MSKCC prognosis; 73% versus 65% were men, and 85% versus 73% were < 65 years of age. All (100%) Asian patients were of Chinese ethnicity. Median duration of exposure was 5.5 mo for Asian and 6.0 mo for non-Asian patients. Among Asian versus non-Asian patients, median progression-free survival (months) was 7.4 versus 7.8 overall, 7.4 versus 4.0 with prior sunitinib, and 5.7 versus 9.2 with prior other anti-VEGFs. Clinical benefit rate was similar between populations: 74.5% (95% CI 61.0–85.3) for Asian patients and 74.7% (95% CI 63.6–83.8) for non-Asian patients. Most patients achieved stable disease as best overall response (Asian, 63.6%; non-Asian, 69.6%). Overall rate of grade 3/4 adverse events appeared similar for Asian (58%) and non-Asian patients (54%).

**Conclusions:**

This RECORD-4 subanalysis demonstrated comparable efficacy and adverse event profiles of second-line everolimus in Asian and non-Asian patients. Efficacy and safety outcomes by prior therapy should be interpreted with caution because of small patient numbers in some subpopulations.

**Trial registration:**

Everolimus as Second-line Therapy in Metastatic Renal Cell.

Carcinoma (RECORD-4); ClinicalTrials.gov identifier: NCT01491672. Registration date: December 14, 2011.

**Electronic supplementary material:**

The online version of this article (10.1186/s12885-018-4091-5) contains supplementary material, which is available to authorized users.

## Background

Everolimus, a mammalian target of rapamycin inhibitor, is a second-line treatment option for patients with metastatic renal cell carcinoma (mRCC) refractory to vascular endothelial growth factor (VEGF) receptor inhibitors [1, 2]. Approval of everolimus was based on results of the phase 3 RECORD-1 study of patients who had previously received sunitinib, sorafenib, or both [3]. The phase 2 RECORD-4 study was subsequently designed to assess everolimus as a purely second-line therapy in patients with mRCC [4]. Because 41% of RECORD-4 patients were from China, and some differences in responses to certain targeted agents between Chinese and Western patients have been reported [5], this subanalysis evaluated outcomes in Asian patients compared with non-Asian patients.

## Methods

The RECORD-4 primary analysis has been published [4]. Briefly, 134 adult patients with confirmed clear cell mRCC (RECIST v1.0) and a Karnofsky performance status of ≥70% were enrolled into 1 of 3 cohorts based on their previous first-line therapy: sunitinib, other anti-VEGF agents (sorafenib, bevacizumab, pazopanib, tivozanib, and axitinib), or cytokines. Patients received everolimus 10 mg/d until disease progression, unacceptable toxicity, treatment discontinuation, or death. Dose reduction to 5 mg/d was permitted to manage adverse events (AEs). The primary end point was progression-free survival (PFS) per local radiologic assessment. Median PFS was 7.8 months (95% confidence interval [CI] 5.7–11.0) in the overall population, 5.7 months (95% CI 3.7–11.3) in the first-line sunitinib cohort, 7.8 months (95% CI 5.7–11.0) in the first-line other anti-VEGF therapy cohort, and 12.9 months (95% CI 2.6–not estimable [NE]) in the first-line cytokine-based therapy cohort.

## Results

### Patients

In the Asian (*n* = 55) and non-Asian (*n* = 79) populations, respectively, 98% and 84% had good/intermediate Memorial Sloan Kettering Cancer Center (MSKCC) prognosis, 73% and 65% were men, and 85% and 73% were < 65 years of age (Additional file 1: **Table S1**). Median duration of exposure was 5.5 and 6.0 months for Asian and non-Asian patients, respectively.

### Efficacy

**Fig. 1:**
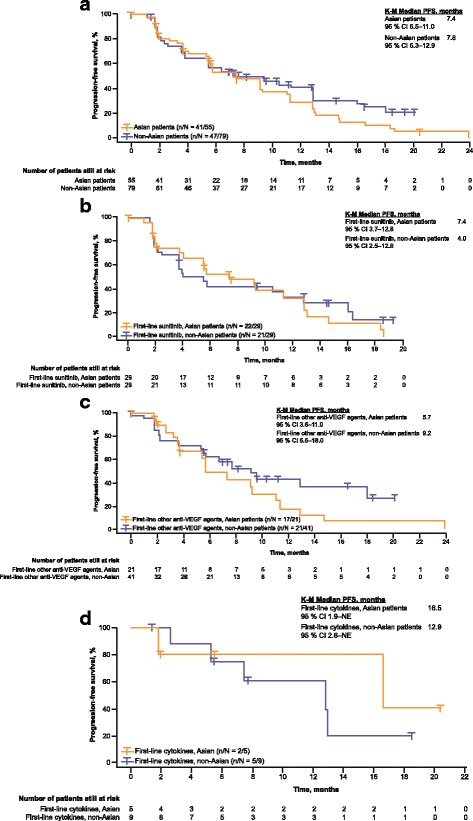
Progression-free survival (PFS) in the Asian and non-Asian populations (**a**), and by first-line treatment cohorts in the Asian and non-Asian populations for (**b**) sunitinib, (**c**) other anti-VEGF agents, and (**d**) cytokines. CI=confidence interval; K-M=Kaplan-Meier; NE=not estimable; PFS=progression-free survival; VEGF=vascular endothelial growth factor

Median PFS was 7.4 months (95% CI 5.5–11.0) for Asian patients (*n* = 55) and 7.8 months (95% CI 5.3–12.9) for non-Asian patients (*n* = 79) (Fig. 1a). In the first-line sunitinib cohort, median PFS was 7.4 months (95% CI 3.7–12.8) in the Asian population (*n* = 29) and 4.0 months (95% CI 2.5–12.9) in the non-Asian population (*n* = 29) (Fig. 1b). In the first-line other anti-VEGF agents cohort, median PFS was 5.7 months (95% CI 3.6–11.0) in the Asian population (*n* = 21) and 9.2 months (95% CI 5.5–18.0) in the non-Asian population (*n* = 41) (Fig. 1c). In the first-line cytokines cohort, median PFS was 16.5 months (95% CI 1.9–NE) in the Asian population (*n* = 5) and 12.9 months (95% CI 2.6–NE) in the non-Asian population (*n* = 9) (Fig. 1d). Best overall response was stable disease for 64% and 70% of Asian (*n* = 35) and non-Asian patients (*n* = 55), respectively (Additional file 2: **Table S2**). Clinical benefit rate (complete response + partial response + stable disease) was 75% (95% CI 61.0–85.3) for Asian patients (*n* = 41) and 75% (95% CI 63.6–83.8) for non-Asian patients (*n* = 51).

### Adverse event profile

The overall rate of grade 3 and 4 AEs (irrespective of relationship to study drug) was 58% and 54% for Asian and non-Asian patients, respectively (Additional file 3: **Table S3**). The most common grade 3 and 4 AEs were anemia (7%), decreased hemoglobin level (6%), hypertriglyceridemia (6%), mouth ulceration (6%), proteinuria (6%), respiratory failure (6%), and stomatitis (5%) among Asian patients and anemia (17%), hyperglycemia (5%), and stomatitis (5%) among non-Asian patients. In the Asian and non-Asian populations, respectively, 22 (40%) and 35 (45%) patients required dose adjustment or study drug interruption to manage AEs. Eleven patients (20%) in the Asian population and 13 patients (17%) in the non-Asian population discontinued treatment because of AEs. Causes of on-treatment deaths in the Asian population were disease progression (*n* = 3), respiratory failure (*n* = 2), and multiorgan failure (*n* = 1) and in the non-Asian population were multiorgan failure (*n* = 2), cardiopulmonary failure (*n* = 1), disease progression (*n* = 1), sepsis (*n* = 1), sudden death (*n* = 1), and unknown cause (*n* = 1).

## Discussion

There have been some reported differences in responses to certain targeted agents between Chinese and Western patients with RCC [5]. However, our findings support the comparable efficacy of everolimus in Asian and non-Asian patients with RCC. In this RECORD-4 subanalysis of second-line everolimus, median PFS was 7.4 and 7.8 months in Asian and non-Asian patients, respectively, and the clinical benefit rate was 75% in both patient populations. These findings suggest that second-line everolimus has comparable efficacy in Asian and non-Asian patients with RCC. These results are supported by a phase 1b study of everolimus in 64 Chinese mRCC patients who were intolerant to, or progressed on, prior VEGFR-TKI therapy, in which median PFS was 6.9 months and the clinical benefit rate was 66% [6]. Median PFS was comparatively shorter (4.9 months) in the pivotal phase 3 RECORD-1 trial of everolimus in VEGFR-TKI pretreated mRCC patients from centers across Australia, Canada, Europe, the USA, and Japan [3]. However, patients in RECORD-1 were more heavily pretreated, having received a median of 2 prior antineoplastic therapies, and had a poorer risk profile per MSKCC criteria, which may negatively affect outcomes in RECORD-1 compared with RECORD-4 [3, 4].

Five targeted drugs (pazopanib, everolimus, axitinib, sorafenib, and sunitinib) are currently approved for the treatment of advanced RCC in China, and everolimus and axitinib carry the highest level of evidence for second-line treatment after failure of first-line TKI in Chinese and Asia consensus treatment guidelines [7–9]. Thus, results of our study are important to physicians who treat these populations of patients, and support the use of everolimus following progression on first-line TKI therapy in Asian patients with mRCC. It is important that outcomes by prior therapy should be interpreted with caution because of the small patient numbers in some subpopulations.

## Conclusions

In conclusion, second-line everolimus had comparable efficacy and safety in Asian and non-Asian patients with mRCC.

## Additional files

Additional files 1: **Table S1** Baseline demographics and disease characteristics of Asian and non-Asian patients in the overall population and in the first-line therapy cohorts. (DOCX 15 kb)

Additional files 2: **Table S2** Tumor responses (RECIST v1.0) of Asian and non-Asian patients in the overall population and in the first-line therapy cohorts. (DOCX 13 kb)

Additional files 3: **Table S3** Grade 3 and 4 adverse events reported by Asian and non-Asian patients in the overall population and in the first-line therapy cohorts. (DOCX 13 kb)

## Abbreviations

AEs: Adverse events
mRCC: Metastatic renal cell carcinoma
MSKCC: Memorial Sloan Kettering Cancer Center
PFS: Progression-free survival
RECIST: Response Evaluation Criteria In Solid Tumors
VEGF: Vascular endothelial growth factor
