# Supplementary material for: RECORD-4 multicenter phase 2 trial of second-line everolimus in patients with metastatic renal cell carcinoma: Asian versus non-Asian population subanalysis
Source: BMC Cancer. 2018 Feb 17;18:195. doi: 10.1186/s12885-018-4091-5 (PMC5816475; doi:10.1186/s12885-018-4091-5)
Supplement: Supplementary file 1 — Table S1 Baseline demographics and disease characteristics of Asian and non-Asian patients in the overall population and in the first-line therapy cohorts. (DOCX 15 kb) [file 12885_2018_4091_MOESM1_ESM.docx]

**Table S1. Baseline demographics and disease characteristics**

|  | **Overall Population** | | **Prior Therapy** | | | | | |
| --- | --- | --- | --- | --- | --- | --- | --- | --- |
|  |  |  | **Sunitinib** | | **Other Anti-VEGF Agents** | | **Cytokines** | |
|  | **Asian**  ***n* = 55** | **Non-Asian**  ***n* = 79** | **Asian**  ***n* = 29** | **Non-Asian**  ***n* = 29** | **Asian**  ***n* = 21** | **Non-Asian**  ***n* = 41** | **Asian**  ***n* = 5** | **Non-Asian**  ***n* = 9** |
| Median age, y (range) | 55  (18–78) | 60  (23–79) | 55  (23–72) | 62  (23–79) | 52  (18–78) | 59  (23–74) | 60  (39–69) | 63  (48–76) |
| <65 y, *n* (%) | 47 (85) | 58 (73) | 26 (90) | 21 (72) | 17 (81) | 32 (78) | 4 (80) | 5 (56) |
| Sex, n (%)  Men  Women | 40 (73)  15 (27) | 51 (65)  28 (35) | 23 (79)  6 (21) | 20 (69)  9 (31) | 15 (71)  6 (29) | 25 (61)  16 (39) | 2 (40)  3 (60) | 6 (67)  3 (33) |
| MSKCC prognosis,^†^ *n* (%) |  |  |  |  |  |  |  |  |
| Favorable | 40 (73) | 30 (38) | 20 (69) | 12 (41) | 15 (71) | 16 (39) | 5 (100) | 2 (22) |
| Intermediate | 14 (25) | 36 (46) | 8 (28) | 11 (38) | 6 (29) | 18 (44) | 0 (0) | 7 (78) |
| Poor | 1 (2) | 13 (17) | 1 (3) | 6 (21) | 0 (0) | 7 (17) | 0 (0) | 0 (0) |
| Median time since RCC diagnosis, months | 25.1 | 41.4 | 24.9 | 46.7 | 28.8 | 40.9 | 29.5 | 41.8 |
| Median duration of exposure, months | 5.5 | 6.0 | 5.8 | 4.0 | 5.5 | 6.5 | 7.1 | 9.6 |

MSKCC=Memorial Sloan Kettering Cancer Center; RCC=renal cell carcinoma; VEGF=vascular endothelial growth factor.

^†^Patients in the favorable group have no risk factors, patients in the intermediate group have 1 risk factor, and patients in the poor group have 2 or 3 risk factors.
