# Supplementary material for: RECORD-4 multicenter phase 2 trial of second-line everolimus in patients with metastatic renal cell carcinoma: Asian versus non-Asian population subanalysis
Source: BMC Cancer. 2018 Feb 17;18:195. doi: 10.1186/s12885-018-4091-5 (PMC5816475; doi:10.1186/s12885-018-4091-5)
Supplement: Supplementary file 2 — Table S2 Tumor responses (RECIST v1.0) of Asian and non-Asian patients in the overall population and in the first-line therapy cohorts. (DOCX 13 kb) [file 12885_2018_4091_MOESM2_ESM.docx]

**Table S2. Tumor response (RECIST v1.0)**

|  | **Overall Population** | | **Prior Therapy** | | | | | |
| --- | --- | --- | --- | --- | --- | --- | --- | --- |
|  |  |  | **Sunitinib** | | **Other Anti-VEGF Agents** | | **Cytokines** | |
|  | **Asian**  ***n* = 55** | **Non-Asian**  ***n* = 79** | **Asian**  ***n* = 29** | **Non-Asian**  ***n* = 29** | **Asian**  ***n* = 21** | **Non-Asian**  ***n* = 41** | **Asian**  ***n* = 5** | **Non-Asian**  ***n* = 9** |
| Best overall response, *n* (%) |  |  |  |  |  |  |  |  |
| Partial response | 6 (11) | 4 (5) | 2 (7) | 2 (7) | 3 (14) | 0 (0) | 1 (20) | 2 (22) |
| Stable disease | 35 (64) | 55 (70) | 19 (66) | 18 (62) | 13 (62) | 32 (78) | 3 (60) | 5 (56) |
| Progressive disease | 9 (16) | 13 (17) | 6 (21) | 9 (31) | 2 (10) | 4 (10) | 1 (20) | 0 (0) |
| Unknown | 5 (9) | 6 (8) | 2 (7) | 0 (0) | 3 (14) | 4 (10) | 0 (0) | 2 (22) |
| Missing | 0 (0) | 1 (1) | 0 (0) | 0 (0) | 0 (0) | 1 (2) | 0 (0) | 0 (0) |
| ORR,^†^ *n* (95% CI) | 6  (4.1–22.2) | 4  (1.4–12.5) | 2  (0.8–22.8) | 2  (0.8–22.8) | 3  (3.0–36.3) | 0  (0.0–8.6) | 1  (0.5–71.6) | 2  (2.8–60.0) |
| CBR,^‡^ *n* (95% CI) | 41  (61.0–85.3) | 59  (63.6–83.8) | 21  (52.8–87.3) | 20  (49.2–84.7) | 16  (52.8–91.8) | 32  (62.4–89.4) | 4  (28.4–99.5) | 7  (40.0–97.2) |

CBR=clinical benefit rate; CI=confidence interval; ORR=overall response rate; VEGF=vascular endothelial growth factor.

^†^ORR, complete response + partial response.

^‡^CBR, complete response + partial response + stable disease.
