# Supplementary material for: RECORD-4 multicenter phase 2 trial of second-line everolimus in patients with metastatic renal cell carcinoma: Asian versus non-Asian population subanalysis
Source: BMC Cancer. 2018 Feb 17;18:195. doi: 10.1186/s12885-018-4091-5 (PMC5816475; doi:10.1186/s12885-018-4091-5)
Supplement: Supplementary file 3 — Table S3 Grade 3 and 4 adverse events reported by Asian and non-Asian patients in the overall population and in the first-line therapy cohorts. (DOCX 13 kb) [file 12885_2018_4091_MOESM3_ESM.docx]

**Table S3. Grade 3 and 4 adverse events**^†^

|  | **Overall Population**^‡^ | | **Prior Therapy** | | | | | |
| --- | --- | --- | --- | --- | --- | --- | --- | --- |
|  |  |  | **Sunitinib** | | **Other Anti-VEGF** | | **Cytokines** | |
|  | **Asian**  ***n* = 55** | **Non-Asian**  ***n* = 78** | **Asian**  ***n* = 29** | **Non-Asian**  ***n* = 29** | **Asian**  ***n* = 21** | **Non-Asian**  ***n* = 40** | **Asian**  ***n* = 5** | **Non-Asian**  ***n* = 9** |
| Overall, *n* (%) | 32 (58) | 42 (54) | 17 (59) | 15 (52) | 12 (57) | 20 (50) | 3 (60) | 7 (78) |
| Anemia | 4 (7) | 13 (17) | 4 (14) | 2 (7) | 0 (0) | 7 (18) | 0 (0) | 4 (44) |
| Decreased hemoglobin level | 3 (6) | — | 2 (7) | — | 1(5) | — | 0 (0) | — |
| Hypertriglyceridemia | 3 (6) | 3 (4) | 3 (10) | 0 (0) | 0 (0) | 3 (8) | 0 (0) | 0 (0) |
| Mouth ulceration | 3 (6) | — | 1 (3) | — | 2 (10) | — | 0 (0) | — |
| Proteinuria | 3 (6) | 1 (1) | 3 (10) | 1 (3) | 0 (0) | 0 (0) | 0 (0) | 0 (0) |
| Respiratory failure | 3 (6) | 1 (1) | 1 (3) | 0 (0) | 2 (10) | 1 (3) | 0 (0) | 0 (0) |
| Stomatitis | 3 (5) | 4 (5) | 1 (3) | 2 (7) | 1 (5) | 1 (3) | 1(20) | 1 (11) |
| Hyperglycemia | 2 (4) | 4 (5) | 1 (3) | 1 (3) | 1 (5) | 3 (8) | 0 (0) | 0 (0) |

VEGF = vascular endothelial growth factor.

^†^Reported in >5% of either the overall Asian or the non-Asian population.

^‡^One patient died before treatment initiation and was excluded from the safety analysis.
